# Supplementary figures and images for: Yield, lodging, and water use efficiency of Tef [Eragrostis tef (zucc) Trotter] in response to carbonized rice husk application under variable moisture condition
Source: PLoS One. 2024 Mar 7;19(3):e0298416. doi: 10.1371/journal.pone.0298416 (PMC10919715; doi:10.1371/journal.pone.0298416)

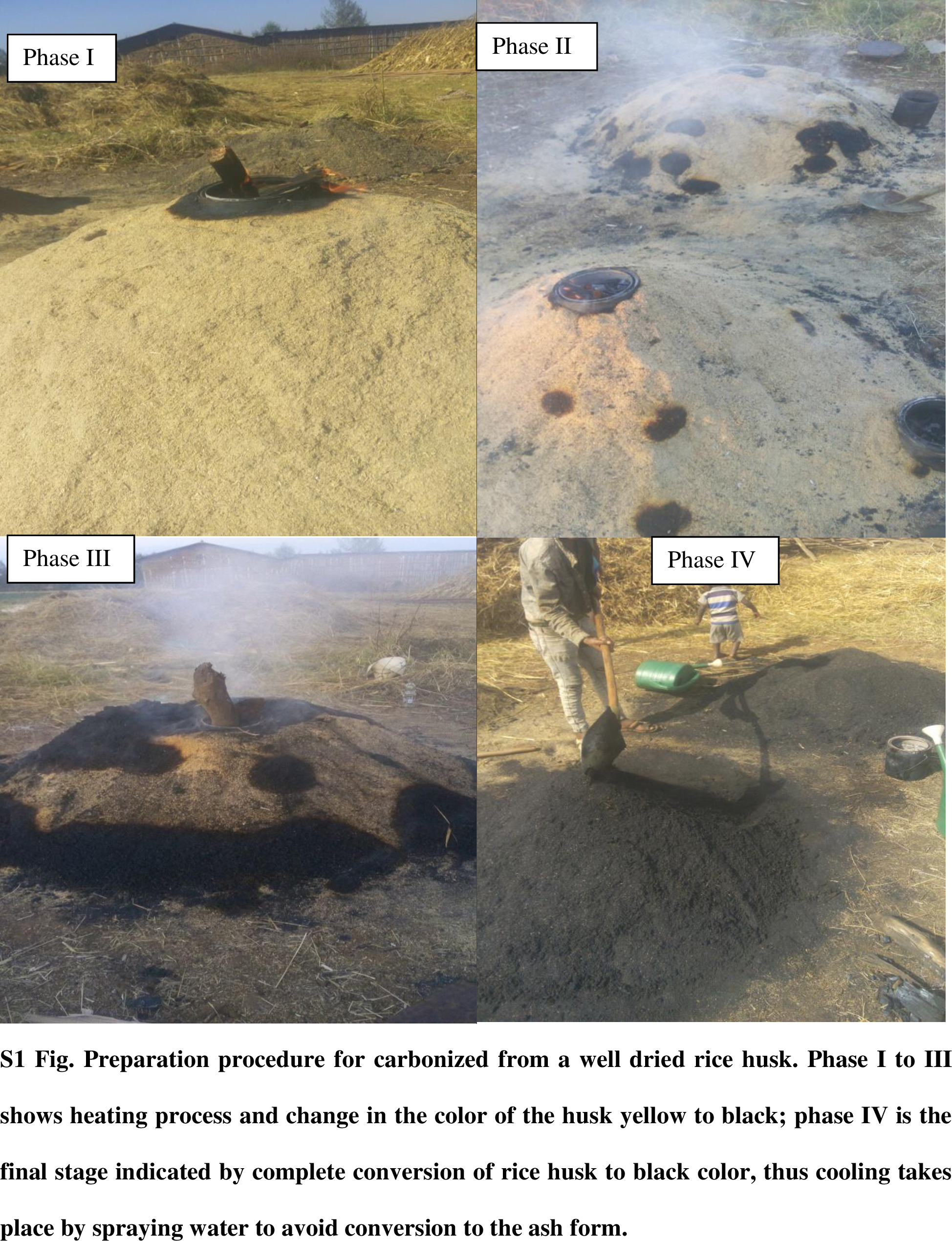

Supplement: S1 Fig — Phase I to III shows heating process and change in the color of the husk yellow to black; phase IV is the final stage indicated by complete conversion of rice husk to black color, thus cooling takes place by spraying water to avoid conversion to the ash form. (TIF) [file pone.0298416.s001.tif]

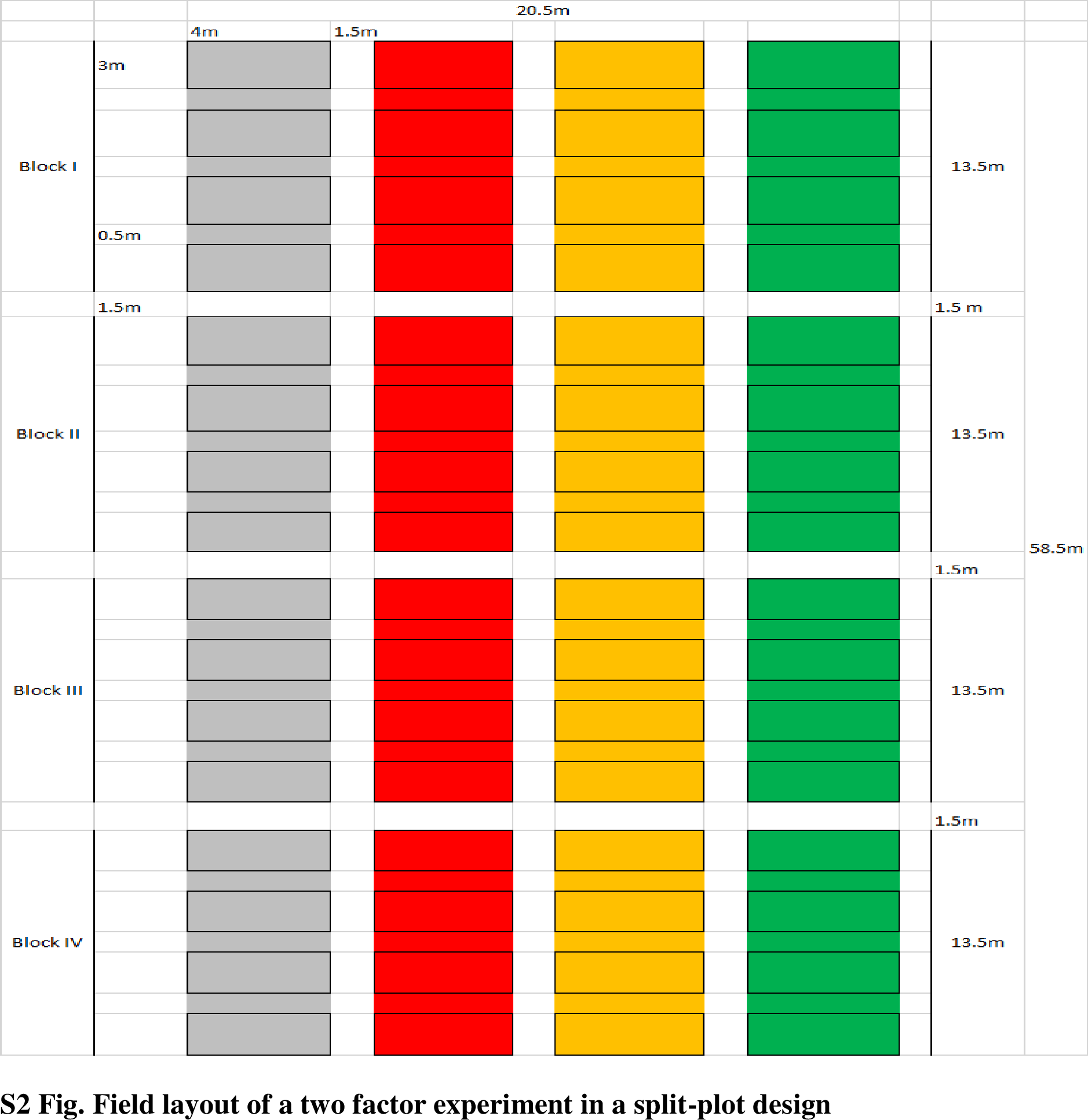

Supplement: S2 Fig — (TIF) [file pone.0298416.s002.tif]

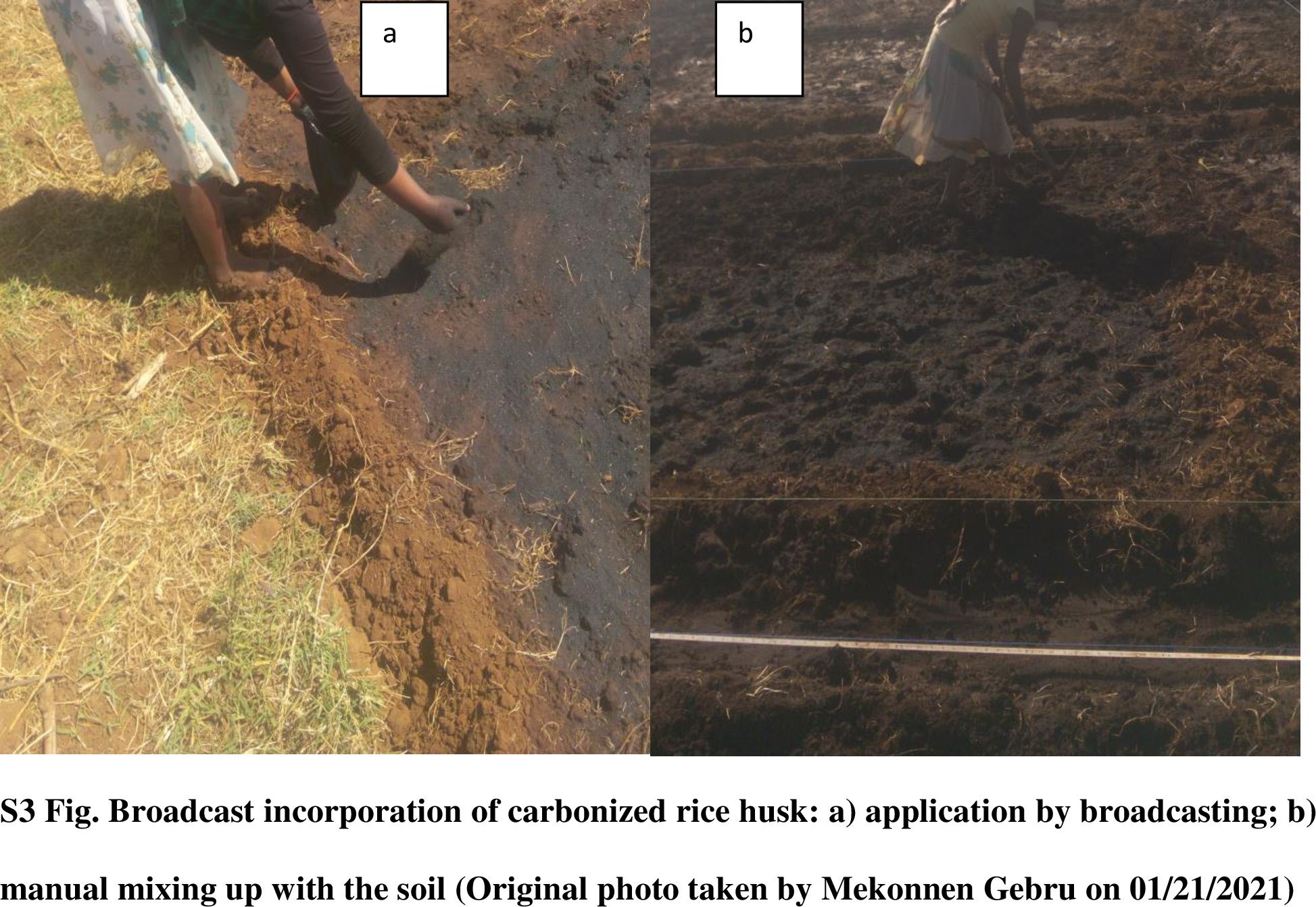

Supplement: S3 Fig — a) Application by broadcasting; b) manual mixing up with the soil. (TIF) [file pone.0298416.s003.tif]
