# Supplementary material for: Yield, lodging, and water use efficiency of Tef [Eragrostis tef (zucc) Trotter] in response to carbonized rice husk application under variable moisture condition
Source: PLoS One. 2024 Mar 7;19(3):e0298416. doi: 10.1371/journal.pone.0298416 (PMC10919715; doi:10.1371/journal.pone.0298416)
Supplement: Attachment — Submitted filename: PONE-D-23-13722_reviewer_R1.pdf [file pone.0298416.s006.pdf]

PLOS ONE

Yield, lodging, and water use efficiency of Tef [Eragrostis tef (zucc) Trotter] in response to carbonized rice husk application under variable moisture condition

--Manuscript Draft--

**Manuscript Number:**	PONE-D-23-13722
**Article Type:**	Research Article
**Full Title:**	Yield, lodging, and water use efficiency of Tef [Eragrostis tef (zucc) Trotter] in response to carbonized rice husk application under variable moisture condition
**Short Title:**	Tef [Eragrostis tef (zucc) Trotter] response to carbonized rice husk under variable soil moisture condition
**Corresponding Author:**	Mekonnen Gebru Tekle, PhD candidate<br>Bahir Dar University College of Agriculture and Environmental Sciences<br>Bahir Dar, Amhara ETHIOPIA
**Keywords:**	carbonized rice husk; silicon; moisture depletion level; tef crop; lodging
**Abstract:**	Terminal drought and lodging are among the major yield limiting factors for tef cultivation in the highly weathered soils of the Ethiopian highlands. Therefore, a study was conducted to assess the yield and lodging responses of tef to varying soil moisture depletion levels (MDL) and the application of carbonized rice husk (CRH). A two-year 4×4 factorial experiment with 20, 35, 55, and 75% moisture depletion levels and 0, 291, 582, and 873 kg ha-1 of CRH was laid out in a split-plot design, with each treatment replicated four times. The pooled mean ANOVA result showed LAI and LI were not significantly influenced by both the main and interaction effects of MDL and CHR ($p > 0.05$); however, the individual year ANOVA showed that both LI and LAI were influenced by the interaction of MDL and CRH ($p<0.05$) in 2021 and 2022, respectively. The lowest LI (19.7%) was obtained from the application of 873 kg CRH ha-1, followed by 20.6% from 582 kg CHR ha-1 in 2022. A 20.7% LI reduction was recorded in 2022 compared to 2021. Tef PH and NTPP were significantly affected by the main effect of MDL at $p<0.05$ and $p<0.01$, respectively, but not by the main and interaction effects of CRH and its interaction with MDL. The effect of MDL was significant on tef HI ($p<0.01$) but not on traits including grain yield, straw yield and WUE. In conclusion, a significantly higher maximum HI of tef (33.8%) was obtained from MDL (55%) and the lowest LI (31.9%) was obtained, though there was a non-significant improvement in GY. Alternative silicon fertilizer sources, which are more economically effective, need to be considered in the future research.
**Order of Authors:**	Mekonnen Gebru Tekle, PhD candidate<br>Getachew Alemayehu Damot, PhD<br>Yayeh Bitew Bantie, PhD
**Additional Information:**	
**Question**	**Response**
**Financial Disclosure**<br><br>Enter a financial disclosure statement that describes the sources of funding for the work included in this submission. Review the submission guidelines for detailed requirements. View published research articles from *PLOS ONE* for specific examples.<br><br>This statement is required for submission and **will appear in the published article** if	This research was funded by the local government, partly by the Ministry of Education and the College of Agriculture and Environmental Sciences of Bahir Dar University.

the submission is accepted. Please make sure it is accurate.<br><br>**Unfunded studies**<br>Enter: *The author(s) received no specific funding for this work.*<br><br>**Funded studies**<br>Enter a statement with the following details:<br>• Initials of the authors who received each award<br>• Grant numbers awarded to each author<br>• The full name of each funder<br>• URL of each funder website<br>• Did the sponsors or funders play any role in the study design, data collection and analysis, decision to publish, or preparation of the manuscript?<br>• **NO** - Include this sentence at the end of your statement: *The funders had no role in study design, data collection and analysis, decision to publish, or preparation of the manuscript.*<br>• **YES** - Specify the role(s) played.<br><br>* typeset	
**Competing Interests**<br><br>Use the instructions below to enter a competing interest statement for this submission. On behalf of all authors, disclose any competing interests that could be perceived to bias this work—acknowledging all financial support and any other relevant financial or non-financial competing interests.<br><br>This statement is required for submission and **will appear in the published article** if the submission is accepted. Please make sure it is accurate and that any funding sources listed in your Funding Information later in the submission form are also declared in your Financial Disclosure statement.<br><br>View published research articles from *PLOS ONE* for specific examples.	We the authors of this manuscript have declared that no competing interests exist.

**NO authors have competing interests**<br><br>Enter: *The authors have declared that no competing interests exist.*<br><br>**Authors with competing interests**<br><br>Enter competing interest details beginning with this statement:<br><br>*I have read the journal's policy and the authors of this manuscript have the following competing interests: [insert competing interests here]*<br><br>* typeset	
**Ethics Statement**<br><br>Enter an ethics statement for this submission. This statement is required if the study involved:<br><br>• Human participants<br>• Human specimens or tissue<br>• Vertebrate animals or cephalopods<br>• Vertebrate embryos or tissues<br>• Field research<br><br>Write "N/A" if the submission does not require an ethics statement.<br><br>General guidance is provided below. Consult the submission guidelines for detailed instructions. **Make sure that all information entered here is included in the Methods section of the manuscript.**	N/A

**Format for specific study types**<br><br>**Human Subject Research (involving human participants and/or tissue)**<br>• Give the name of the institutional review board or ethics committee that approved the study<br>• Include the approval number and/or a statement indicating approval of this research<br>• Indicate the form of consent obtained (written/oral) or the reason that consent was not obtained (e.g. the data were analyzed anonymously)<br><br>**Animal Research (involving vertebrate animals, embryos or tissues)**<br>• Provide the name of the Institutional Animal Care and Use Committee (IACUC) or other relevant ethics board that reviewed the study protocol, and indicate whether they approved this research or granted a formal waiver of ethical approval<br>• Include an approval number if one was obtained<br>• If the study involved *non-human primates*, add *additional details* about animal welfare and steps taken to ameliorate suffering<br>• If anesthesia, euthanasia, or any kind of animal sacrifice is part of the study, include briefly which substances and/or methods were applied<br><br>**Field Research**<br><br>Include the following details if this study involves the collection of plant, animal, or other materials from a natural setting:<br>• Field permit number<br>• Name of the institution or relevant body that granted permission	
**Data Availability**<br><br>Authors are required to make all data underlying the findings described fully available, without restriction, and from the time of publication. PLOS allows rare exceptions to address legal and ethical concerns. See the PLOS Data Policy and FAQ for detailed information.	Yes - all data are fully available without restriction

A Data Availability Statement describing where the data can be found is required at submission. Your answers to this question constitute the Data Availability Statement and **will be published in the article**, if accepted.<br><br>**Important:** Stating 'data available on request from the author' is not sufficient. If your data are only available upon request, select 'No' for the first question and explain your exceptional situation in the text box.<br><br>Do the authors confirm that all data underlying the findings described in their manuscript are fully available without restriction?	
**Describe where the data may be found in full sentences. If you are copying our sample text, replace any instances of XXX with the appropriate details.**<br><br>• If the data are **held or will be held in a public repository**, include URLs, accession numbers or DOIs. If this information will only be available after acceptance, indicate this by ticking the box below. For example: *All XXX files are available from the XXX database (accession number(s) XXX, XXX.)*.<br>• If the data are all contained **within the manuscript and/or Supporting Information files**, enter the following: *All relevant data are within the manuscript and its Supporting Information files.*<br>• If neither of these applies but you are able to provide **details of access elsewhere**, with or without limitations, please do so. For example:<br><br>*Data cannot be shared publicly because of [XXX]. Data are available from the XXX Institutional Data Access / Ethics Committee (contact via XXX) for researchers who meet the criteria for access to confidential data.*<br><br>*The data underlying the results presented in the study are available from (include the name of the third party*	All relevant data are within the manuscript and its Supporting Information files.

and contact information or URL).<br>• This text is appropriate if the data are owned by a third party and authors do not have permission to share the data.<br><br>* typeset	
Additional data availability information:	Tick here if the URLs/accession numbers/DOIs will be available only after acceptance of the manuscript for publication so that we can ensure their inclusion before publication.

Manuscript

Click here to access/download;Manuscript;Journal - PLOS ONE.docx

Yield, lodging, and water use efficiency of Tef [*Eragrostis tef* (zucc) Trotter] in response to carbonized rice husk application under variable moisture condition

Mekonnen Gebru Tekle[1,2*], Getachew Alemayehu[2¶], Yayeh Bitew[2¶]

[1]Horticulture, College of Agriculture and Natural Resource Management, Wolkite University,

P.O.Box 07, Wolkite, Gurage, Ethiopia

[2]Bahir Dar University, College of Agriculture and Environmental Science, P.O. Box 5501, Bahir

Dar, Ethiopia

**Corresponding author*

E-mail address: mekugebru@gmail.com

[¶]This authors contributed equally to this work

Abstract

Terminal drought and lodging are among the major yield limiting factors for tef cultivation in the highly weathered soils of the Ethiopian highlands. Therefore, a study was conducted to assess the yield and lodging responses of tef to varying soil moisture depletion levels (MDL) and the application of carbonized rice husk (CRH). A two-year 4×4 factorial experiment with 20, 35, 55, and 75% moisture depletion levels and 0, 291, 582, and 873 kg ha^{-1} of CRH was laid out in a split-plot design, with each treatment replicated four times. The pooled mean ANOVA result showed LAI and LI were not significantly influenced by both the main and interaction effects of MDL and CHR ($p > 0.05$); however, the individual year ANOVA showed that both LI and LAI were influenced by the interaction of MDL and CRH ($p<0.05$) in 2021 and 2022, respectively. The lowest LI (19.7%) was obtained from the application of 873 kg CRH ha-1, followed by 20.6% from 582 kg CHR ha^{-1} in 2022. A 20.7% LI reduction was recorded in 2022 compared to 2021. Tef PH and NTPP were significantly affected by the main effect of MDL at $p<0.05$ and $p<0.01$, respectively, but not by the main and interaction effects of CRH and its interaction with MDL. The effect of MDL was significant on tef HI ($p<0.01$) but not on traits including grain yield, straw yield and WUE. In conclusion, a significantly higher maximum HI of tef (33.8%) was obtained from MDL (55%) and the lowest LI (31.9%) was obtained, though there was a non-significant improvement in GY. Alternative silicon fertilizer sources, which are more economically effective, need to be considered in the future research.

Key words: carbonized rice husk, silicon, moisture depletion level, tef

Introduction

Drought and lodging are among the major problems in agricultural crop production in Ethiopia. Drought reduces the yield of crops, restricts growth and photosynthesis, changes morphology, decreases chlorophyll content, and affects ion balance in plants [1]. The extent of the drought effect varies with crop type and growth stage. For instance, legumes are more sensitive than cereals to drought and most of them are more affected at their reproductive stages [2]. Despite the variation in the extent of its effect among various crops, it affects the growth and yield of all type of crops.

The majority of farmers in Ethiopia are engaged in smallholder, dry land agricultural systems [3]. This production system is vulnerable to drought caused by inter-annual rainfall variability and uptake by plants [4]. Many reports also showed the effect of drought on nutrient availability and uptake of plants [5-7]. In recent years, the increase in price in the local and international market for food crops, increased the interest of farmers and the government in producing tef under irrigation. However, heavy fertilization and uncontrolled flooding could result in the lodging of plants and reduction in yield of crops. Therefore, the use of efficient water management along with optimum rate of benefical nutrient technologies is essential since water is becoming scarce [8] and to minimize crop yield loss [9].

Lodging, another yield limiting factor, has been reducing yield and quality of cereal crops [10-11], which is attributed to low assimilation supply during the grain filling stage [12]. Lodging is a serious problem that could generally result in a significant economic loss for the country [13]. A review showed that lodging under natural condition could cause up to 22% Tef yield loss, 35% of 1000-kernel weight, and 51% of grain yield per panicle [14].

A review [14] showed that application of growth hormones like giberelline induces lodging resistance by inhibiting plant hieght in tef. The idea of improving lodging resistance through regulation of plant height was also stated by many other researchers [15-18]. Other organic compounds like paclobutrazol improve the stem strength of wheat by increasing lignin accumulation and its related enzyme activity in the basal second internode [10,19]. However, paclobutrazol caused a significant reduction in plant height [20], which could not be acceptable by farmers, as the straw has multiple benefits for their livelihood. In barley-pea intercropping, mechanical support from barley was found to be useful to reduce the lodging effect of pea [21], but it could be less applicable for tef because of severe resource competition and difficulty of harvesting.

Many other research reports stated the positive effect of silicon fertilization on the yield and lodging resistance [22-23], disease [24], and water stress tolerance [25-26] of various high silicon accumulating monocotyledon crops and sugar cane [27]. Another researcher also found significant effect of silicon in the form of potassium silicate on improving water use efficiency of maize crops [28]. However, some of the low silicon accumulating crops, such as potato [29] and tomato, were also found responsive to silicon. According to [30], the defensive property of silicon can help reduce the use of pesticides. The application of silicon increases light interception [31], increase P availability, and avoid heavy metal toxicity [32]Savant *et al.*, 1999), thus, biomass and grain yield of crops can be maximized. Three foliar applications of Silixol ended in an average yield increment of about 15% in rice [33]. As per [34], silicon transcriptionally regulates sulfur and abscic acid metabolism and delays leaf senescence in barley under combined sulfur deficiency and osmotic stress. Moreover, [33] indicated the regulation of sodium transportation and distribution in maize plants growing in mild salt stress conditions.

Sorghum grown under water stress conditions can provide the highest yield with a synergetic effect of irrigation and increased silicon uptake [35]. The application of silicon in the form of orthosilicic acid (H_4SiO_4) positively affected the growth, yield, and nutrient uptake of rice in tropical zones of Vietnam [36].

The uptake of Si is as high as that of other macronutrients like P, N and Ca in high accumulating crops like rice and sugar cane. Plants absorb Si in the form of orthosilicic acid (H_4SiO_4), which is not very mobile in plants. Three mechanisms of absorption are known for Si uptake: passive, active and exclusion. The first two mechanisms are common in high Si accumulating plant species. Tissue analysis from a wide variety of plants showed that silicon concentrations range from 1 to 100 g Si kg^{-1} of dry weight, depending on plant species. On average monocot plants absorb 50 to 200 kg of Si ha^{-1}. The largest amounts of silicon are absorbed by sugarcane (300–700 kg of Si ha^{-1}), rice (15–300 kg of Si ha^{-1}), and wheat (5–150 kg of Si ha^{-1}) [33].

Although Si is abundant in the earth's crust, its availability is low to supply the need for plants. The upper 20 cm soil layer contains only an average of 0.1 to 1.6 kg Si ha^{-1} as orthosilicic acid [37]. Inorganic silicon sources were used most commonly for research as well as production of cereals, however, they may not be eco-friendly [38] or locally available, so alternative organic sources need to be uncovered. According to [39] stated rice husk as good source of silicon and carbon. According to [40], rice husk ash contains more than 60% of silica. A recent research report also indicated up to 90% silicon content of rice husk [41]. Other researcher [42] found that carbonized rice husk as cost-effective natural fertilizer source for silicon and a capability of improving soil carbon sequestration.

Tef is amongst the major cereal crops widely grown in various climatic and soil conditions in Ethiopia [43]. Its multiple use and wide adaptation make the crop preferred both by the growers and consumers in country. The pan like bread called ‘injera’, which is prepared from tef grain, is the staple cultural food by nearly 75% of the people in the country [44]. In recent years, tef has attracted baking factories from developed countries for making gluten-free food products [45]. The straw has also been used as a livestock feed, cement for the construction of traditional houses. Despite, multiple benefits of tef, its productivity has been hindered by drought [8] and lodging [14], among others. Conversely, the yield and lodging response of tef towards the application of silicon under variable soil moisture condition particularly in an open environment were not verified. Therefore, the objective of this experiment was to assess the water use efficiency, yield, and lodging responses of tef to varying soil moisture content and carbonized rice husk supplement.

Materials and methods

2.1. Site description

A two-year field experiment was conducted to assess the productivity and lodging responses of tef to varying soil moisture content and silicon application in the two dry seasons of 2021 and 2022 at the Bahir Dar University Research Site, Kudmi, Mecha District, Northwest Ethiopia. The site map for the study site is provided below (Fig 1). The study site is located at 11°23'32" N latitude and 37°6'42" E longitude at an elevation of 1983 m.a.s.l. Based on the 30 years of weather data, the mean monthly minimum and maximum temperatures were 10.7 and 27.7 ^{o}C, respectively. The mean annual rainfall was 1768 mm. A summary of mean daily minimum and maximum temperatures and rainfall variability is shown graphically hereunder

(Fig 2). The missing values of weather data were filled by calculating based on the means and variance of the observed data through DSSAT WeatherMan Version 4.8.0.0 [46].

Fig 1. Map of the study site

Fig 2. Mean daily minimum and maximum temperatures and rain weather data for 01/01/1994 to 31/12/2022 (Source: National Meteorological Agency of Ethiopia, Merawi Agro-meteorological station)

Table 1. Pre-planting characteristics of water, soil, and post-planting crop management data

**Irrigation and water quality measurement**							
**Parameter**	**Unit**	**Soil moisture depletion level**				**Critical level**	**Growth stage**
		20	35	55	75		
		%					
I	days	1-2	1-2	1-2	1-2	-	Sowing to 90% emergence
		3-7	3-12	3-12	3-14	-	Emergence to maturity stage
		4.8/5	4.6/6	4.7/7	5.2/8	-	Growing season
IF		17	14	12	11	-	Over season
Ig	mm season^{-1}	1314	1133	1161	1091	-	Over season
**Water Quality range over the two production seasons**							
ECw	µS	88.5-146.0				Low (not saline)	
TDS	ppm	44.9-70.8				Low	
**Soil physicochemical properties by soil profile layer**							
		**0-20 (cm)**	**20-40 (cm)**	**40-60 (cm)**			
ECe	µS	52.5	31.9	32.4		Low (no salinity)	
Soil pH	-	5.0	5.4	5.5		Strongly acidic	
BD	g/cm^3	1.23	1.13	1.10			
OC	%	1.96	1.5	1.38			
**Crop management by crop season (year)**							
		**PD**		**HD**			
Year	1	22/01/2021		06/05/2021			
	2	04/01/2022		(13/04/2022)			

I, irrigation interval; IF, Irrigation frequency; Ig gross irrigation; ECw, Electrical conductivity of water; ECe, Electrical conductivity of soil; PD (HD), planting date and Harvest Date; LGP, Length of growth period; *mean of year 2021/2022

2.2. Experimental planting material

A tef variety called "*Heber-1*" (DZ-Cr-419) was used for the experiments. According to [14], Hiber-1 is a very white seeded variety, which was released in 2017 by Adet Agricultural

Research Centre. It is tolerant to terminal moisture stress, early set, but sensitive to lodging [14]. The variety Hiber-1 is also competitive in yield with Quncho and Kora varieties that are moderately tolerant to lodging. Its altitude and annual rainfall requirements are ranging from 1500-2500 masl and 300-700 mm, respectively. The on-station yield was reported as 2.2-2.7 ton ha^{-1}.

Carbonized rice husk was used as a source of Si. The material was prepared conventionally from well dried rice husk collected from nearby local market. The preparation process takes about 31 hours with the preparation procedure shown in the S1 Fig.

2.3. Experimental design and layout

A 4*4 factorial experiment with four soil moisture depletion levels [20% (no water stress), 35%, 55%, and 75%] and four levels of silicon in the form of carbonized rice husk (0, 291, 582, and 873 kg ha^{-1}) was laid out in a split-plot design with four replications. The treatments for the main and sub-plot factors are presented below in Table 2. The treatments of the sub-plot factor were laid out on a net plot area of 3 m by 4 m (12 m^2). The main-plot factor treatments were laid out on a net experimental plot area of 13.5 m by 4 m (54 m^2). The space between adjacent plots and blocks will be 0.5 m and 1 m, respectively. The gross experimental plot area was 24.5 m by 62.5 m (1531.25 m^2), including a 2 m wide buffer zone. The field layout was provided in S2 Fig.

Table 2. Main- and sub-plot treatments used in a split-plot design

**S.N**	**Main-plot factor levels [MDL (%)]**	**Sub-plot factor levels [CHR (kg ha-1)]**
1	20 (no water stress)	0
2	35	291
3	55	582
4	75	873

MDL, water depletion level; CHR, Carbonized rice husk

2.4. Agronomic practices

To make the soil loose, pre-planting irrigation was applied. Initially, the whole experimental plot was tilled using an ox-driven method. During the second year, each plot was tilled separately using hand-held tools, and clods were broken and levelling was done by hand. Seeds of the Heber-1 variety were sown on the experimental plots at a rate of 15 kg ha^{-1} on a 12 m^2 plot area, which received 18 g of tef seed. The practice of sowing was carried out on well-prepared land using the row planting method at a spacing of 0.2 m.

The Si treatment levels (in the form of carbonized rice husk), were randomly broadcast and incorporated into each experimental (S3 Fig) plot following the standard procedure provided for split plot design [47]. The application was done just before sowing with the broadcast incorporation method and thoroughly mixed with a digging hoe. Each plot received the recommended amounts of nitrogen (40 kg ha^{-1}) and phosphorous (60 kg ha^{-1}) in the form of urea and triple superphosphate (TSP), respectively. For good plant growth, urea was applied in a split application with 1/3 at planting and 2/3 at stem elongation, whereas TSP was applied as a band application at sowing. Other agronomic practices, including weeding and pest control, were done as per the recommendation provided for tef production in the study area [48]. Irrigation treatment was commenced when the seedlings fully emerged (8 DAP). The soil moisture content was monitored with the help of a soil moisture meter (Delta-T device, Cambridge, UK). The soil was refilled to field capacity from the point of depletion of the corresponding treatments.

2.5. Data collection

Plant height (PH) and panicle length (PL) were measured from the same ten plant samples, which were selected randomly from the net plot area; the earlier parameter was measured from the base of the stem to the tip of the panicle, while the later one was measured

from the node, where the first panicle branch emerges, to the panicle tip by a hand-held measuring tape. Leaf area index (LAI) was taken at mid-season stage with the help of a canopy analyser (LI-COR Biosciences, LAI-2250, PCH-4750, made in the USA). The number of tillers per plot was counted at maturity stage from ten randomly selected plants in the net plot area. Plant growth during the period of water deficit was determined by calculating the relative growth rate (RGR) on the basis of PH measured every fortnight (Equation 1).

$$RGR = \frac{PH_1 - PH_o}{PH_o} \qquad (1)$$

where PH_1 and PH_o represent the PH (cm) measured at consecutive periods, with PH_o the initial measurement and PH_1 as the next measurement.

The physiological parameters of water use efficiency (WUE) were calculated from grain yield adjusted for moisture content and the amount of water applied through irrigation (Equation 2). Chlorophyll content was measured every fortnight using a chlorophyll meter (SPAD-502 Plus, KONICA MINOLTA, INC, made in Japan) non-destructively on the leaves of five sample plants.

$$WUE = GY_{adj} / Ia \qquad (2)$$

where WUE is water use efficiency; GYadj is adjusted grain yield (kg ha^{-1}); and I_g is gross applied irrigation (m^3).

Tef grain yield (GY) and above-ground biomass yield (ABY) were measured after harvest, which was carried out by throwing a 1 m^2 quadrant randomly in a net plot area. The measurement of GY was done by taking the weight of the grains with a portable digital balance ±0.01 measurement error and converting it to hectares just after adjusting its moisture content to 12.5% (Equation 3). The BY was measured by weighing the sun-dried above-ground harvested

samples and converting them to kg ha^{-1}. The harvest index (HI) was calculated as the ratio of GY to ABY and expressed as a percentage, whereas the straw yield was considered the difference between ABY and GY. The lodging index was recorded by monitoring the degree of plant stand inclination towards the ground on the whole plot level with a scale of zero to five, where 0 represented 0% and 5 represented 100% plant lodging [49].

$$GY_{adj} = GY_m * \left({(100 - MC)} \middle/ {(100 - 12.5)} \right) \quad (3)$$

Where, GYadj is adjusted grain yield (kg ha^{-1}) at 12.5% moisture content; GY_m is the actual grain yield; MC is the actual moisture content of measured grain yield (%).

2.6. Data analysis

R statistical software version 4.1.1 was used to analyse the data [50]. The Shapiro-Wilk testing approach was used to determine the normality of the data distribution before performing the analysis of variance (ANOVA) [51]. The ANOVA was performed using a statistical package called 'doebioresearch', which was built in version 4.1.3 for split-plot design analysis. When there was a significant difference, the general linear model's process was followed, and means separation was carried out using the least significant difference procedure at the 0.05 probability level. Models from one-, two-, and three-way ANOVA were fitted to the corresponding experimental design. Water depletion level and rate of CHR were treated as fixed variables in the data analysis, while year and replication were treated as random effects.

3. Result and discussion

3.1. Lodging and leaf area indices

The effect of moisture depletion level and silicon rate on the lodging response of tef was presented below (Table 3). The combined analysis result over the two years showed that lodging and leaf area index of tef were not significantly influenced by either the main or interaction of water depletion level and silicon application rate ($p>0.05$). However, based on the individual-year analysis, LI and LAI were significantly ($p<0.05$) influenced by the interaction of MDL and CRH in 2021 and 2022, respectively. The result also showed that LI was significantly ($p<.001$) affected by the interaction of CRH with production years, but not by LAI. The overall mean values of both indices were significantly higher ($p<0.01$) in 2021 than in the 2022 season. The lowest LI of 19.7%, followed by 20.6%, was recorded in 2022 from the application of 873 kg ha^{-1} CHR, and 582 kg ha^{-1}, respectively. Based on the overall mean recorded values over the two years, it showed that the LI of tef had decreased by 20.7% in 2022 as compared to 2021.

The beneficial effect of Si application on lodging of monocots, including tef, was in line with different research results [52-57]. However, our result had a little deviation from [57] in terms of rate, which had a significant effect on tef lodging and thus might be due to the difference in Si source and rate of release that determine the availability and uptake of Si content by plants. The significant variation in LI due to the effect of CRH application over the years could probably be due to the residual effect of CRH, which was supplied in the previous cropping season and causes relatively shorter PH and pre- and post-harvest management practices. The non-significant effect of treatments based on the COY might be due to the slow release of available silicon from CHR.

Table 3. The main and interaction effect of soil moisture depletion level and silicon rate on lodging and leaf area index of tef

Treatment		LI			LAI		
		**2021**	**2022**	**COY**	**2021**	**2022**	**COY**
**MDL (%)**	Control (20)	45.0^{a}	30.9^{a}	38.0^{a}	3.4^{a}	2.6^{a}	2.99^{a}
	35 (W2)	48.8^{a}	24.4^{ab}	36.6^{a}	3.6^{a}	2.0^{a}	2.79^{a}
	55 (W3)	44.1^{a}	22.5^{b}	33.3^{a}	3.7^{a}	2.1^{a}	2.90^{a}
	75 (W4)	46.9^{a}	24.4^{ab}	35.6^{a}	3.1^{a}	2.4^{a}	2.76^{a}
**CRH (0)**	Control (20)	48.8^{a-d}	41.3^{a}	45.0^{a}	3.93^{ab}	2.83^{a}	3.38^{a}
	35	45.0^{a-d}	30.0^{bc}	37.5^{abc}	3.30^{ab}	1.96^{fgh}	2.6368^{abc}
	55	45.0^{a-d}	30.0^{bc}	31.9^{bc}	3.7^{ab}	2.15^{d-g}	2.91^{abc}
	75	45.0^{a-d}	33.75^{ab}	39.4^{ab}	3.2^{ab}	2.511^{a-d}	2.84^{abc}
**CRH (291)**	Control (20)	45.0^{a-d}	30.00^{bc}	37.5^{abc}	3.26^{ab}	2.64^{ab}	2.95^{abc}
	35	37.5^{cd}	30.0^{bc}	33.8^{bc}	3.0^{b}	1.77^{h}	2.40^{bc}
	55	33.8^{d}	26.3^{bcd}	35.6^{abc}	3.1^{b}	2.21^{def}	2.64^{abc}
	75	56.3^{ab}	26.3^{bcd}	41.3^{ab}	2.4^{b}	2.13^{e-h}	2.28^{c}
**CRH (582)**	Control (20)	45.0^{a-d}	26.3^{bcd}	35.6^{abc}	3.8^{ab}	2.512^{a-d}	3.16^{ab}
	35	60.0^{a}	22.5^{cde}	41.3^{ab}	3.92^{ab}	2.23^{def}	3.08^{abc}
	55	52.5^{abc}	15.00^{e}	33.8^{bc}	3.5^{ab}	1.80^{gh}	2.637^{abc}
	75	37.5^{cd}	18.75^{de}	28.1^{c}	4.0^{ab}	2.37^{b-e}	3.19^{ab}
**CRH (873)**	Control (20)	41.3^{bcd}	26.3^{bcd}	33.8^{bc}	2.7^{b}	2.27^{c-f}	2.48^{bc}
	35	52.5^{abc}	15.00^{e}	33.8^{bc}	4.1^{ab}	2.03^{e-h}	3.04^{abc}
	55	45.0^{a-d}	18.75^{de}	31.9^{bc}	4.8^{a}	2.09^{e-h}	3.44a
	75	48.75^{a-d}	18.75^{de}	33.8^{bc}	2.9^{b}	2.60^{abc}	2.73^{abc}
**Significance (ANOVA)**							
**Y**				**			**
**MDL**		ns	ns	ns	ns	ns	ns
**CRH**		ns	ns	ns	ns	ns	ns
**Y*CRH**				***			ns
**Y*MDL**				ns	ns	ns	ns
**MDL*CRH**		*	ns	ns	ns	*	ns
**CV (%)[1]**		25.9	35.4	29.0	42.2	41.6	43.0
**CV (%)[2]**		25.1	27.8	27.4	32.8	11.3	28.8

Y, Year; MDL, moisture depletion level; rate of silicon was in kg ha^{-1}; CRH, carbonized rice husk; Y*CRH, interaction of year and carbonized rice husk; Y*MDL; year*moisture depletion level; MDL*CRH, interaction of moisture depletion and carbonized rice husk; ANOVA, analysis of variance; CV, coefficient of variation; ns, non-significant; **, means are significantly different at p<0.001; ** means significantly different at p<0.01; *, means are significantly different at P<0.05

3.2. Plant height

The response of tef plant height to different soil moisture depletion levels, silicon rates, and their interactions is provided in Table 4. Plant height was significantly (P<0.05) affected by the main effect of MDL. However, it was not influenced by the main effect of CRH and its interaction with MDL (P>0.05). The maximum PH (102.8 cm) was measured from the 20% MDL treatment, but it was not significantly different from the treatment that received irrigation at 35% MDL, which provided a PH of 101.0 cm. There was a 5.7% difference in PH between the 20 and 75% MDL. The smallest difference (0.1%) was found between 55% and 75% MDL. A separate analysis of the yearly plant height result showed the presence of variation across production seasons. The mean plant height (112.4 cm), which was measured in 2021, was significantly (p<0.01) higher than the PH (86.8 cm) of 2022. The difference in PH among water depletion levels might be due to moisture stress that occurred during the early stages of crop development. This was in agreement with the report that showed a stunted growth of field crop plants under critical soil moisture conditions [58].

Table 4. The effect of soil water depletion and the application of silicon on tef plant height (cm)

**Treatments**		**CRH (kg ha^{-1})**							
		**0**	**291**	**582**	**873**	**Mean**	**SE**	**LSD.05**	**CV (%)**
**MDL (%)**	Control (20)	102.3^{a}	102.3^{a}	102.2^{a}	104.3^{a}	**102.8^{a}**			
	35 (W2)	99.5^{a}	99.4^{a}	101.7^{a}	103.4^{a}	**101.0^{ab}**			
	55 (W3)	97.3^{a}	100.3^{a}	97.6^{a}	94.5^{a}	**97.4^{b}**			
	75 (W4)	97.4a	94.8^{a}	98.1^{a}	98.8^{a}	**97.3^{b}**			
	**Mean**	**99.1**	**99.2**	**99.9**	**100.2**	**99.6**	**1.21**	**ns**	**5.0**
	**SE**	**2.42**	**2.42**	**2.42**	**2.42**	**1.21**			
	**LSD.05**	**ns**	**ns**	**ns**	**ns**	**11.8**			
**2021**	Control (20)	113.6^{ab}	110.7^{ab}	111.4^{ab}	113.2^{ab}	**112.2^{a}**	**2.14**		
	35	111.1^{ab}	113.1^{ab}	114.6^{ab}	114.0^{ab}	**113.2^{a}**	**2.14**		
	55	113.4^{ab}	114.8^{a}	111.9^{ab}	109.1^{b}	**112.3^{a}**	**2.14**		
	75	109.8^{ab}	111.5^{ab}	113.2^{ab}	113.5^{ab}	**112.0^{a}**	**2.14**		
	**Mean**	**112.0**	**112.5**	**112.8**	**112.4**	**112.4^{a}**	**1.07**	**ns**	**3.5**
	**SE**	**2.14**	**2.14**	**2.14**	**2.14**	**1.07**			
	**LSD.05**	**ns**	**ns**	**ns**	**ns**	**ns**			
	**CV (%)**					**4.782**			
**2022**	Control (20)	91.1^{a-d}	94^{ab}	93^{abc}	95.4^{a}	**93.4^{a}**			
	35	87.9^{a-f}	85.8^{b-g}	88.9^{a-e}	92.8^{abc}	**88.8^{ab}**			
	55	81.1^{efg}	85.8^{b-g}	83.3^{d-g}	79.9^{fg}	**82.5^{b}**			
	75	85.0^{c-g}	78.1^{g}	83.1^{d-g}	84.1^{d-g}	**82.6^{b}**			
	**Mean**	**86.3**	**85.9**	**87.1**	**88.0**	**86.8^{b}**	**1.75**	**ns**	
	**SE**	**3.5**	**3.5**	**3.5**	**3.5**	**1.75**			
	**LSD.05**	**ns**	**ns**	**ns**	**ns**	**8.47**			
	**CV (%)**								
**Significance (ANOVA)**									
**Year**		**	**	**	**	**			
**Y*MDL**						.			
**Y*CRH**		**ns**	**ns**	**ns**	**ns**	**ns**			
**MDL*CRH**		**ns**	**ns**	**ns**	**ns**	**ns**			
**Y*MDL*CRH**		**ns**	**ns**	**ns**	**ns**	**ns**			
**CV (%)**						**8.4**			

MDL, water Depletion level; COY, Combined analysis over years; SE, standard error; LSD.05, least significance difference at 0.05 level of significance; CV, Coefficient of variation; Y, Year; Y*MDL, interaction of year and water depletion level; Y*CRH, interaction of year and silicon rate; MDL*CRH, interaction of water depletion level and CRH rate; Y*MDL*CRH, interaction of year, soil moisture depletion level, and CRH; ns, non-significance difference

3.3. Relative growth rate

The effect of water depletion level on the relative growth rate of the tef crop was presented hereunder (Figure 3). The growth rate curve for all four treatments followed a similar trend. Initially, the RGR was slow, then increased sharply between 43 and 71 DAP, and then decreased with a similar pattern. Despite the slow start of RGR from the control treatment (20% MDL), it showed the fastest increment during the second period as compared to the other treatments. The fourth treatment (75% MDL) had shown relatively the fastest RGR of any other treatment, but it slowed during the second phase of growth. The maximum RGR (1.66) was observed from 20% MDL, while the lowest one (1.36) was observed from 75% MDL.

Figure 3. The effect of water depletion level on the relative growth rate of tef

3.3. Panicle length

Panicle length is considered among the most important lodging and yield determinants. The result indicating the main and interaction effects of MDL and CRH over individual and combined analyses over years is presented in Table 5. This trait responded significantly ($p<0.1$) to different water depletion levels, but the effect of different CRH application rates did not influence PL significantly ($p > 0.05$). The interaction of both the main and sub-plot factors also did not significantly ($P>0.05$) affect PL. The overall mean PL measured in 2021 was significantly higher than the value measured in 2022 ($p<0.01$). The mean PL collected in 2021 was 30.3% higher than the mean PL in 2022. This might be due to the difference in pre- and post-harvest crop management and weather conditions [59].

Table 5. The effect of water depletion level and silicon rate on the panicle length of tef

		**COY (PL)**							
		**0**	**291**	**582**	**873**	**Mean**	**SE**	**LSD.05**	**CV (%)**
	Control (20)	37.8^{a}	38.7^{a}	38.2^{a}	38.8^{a}	**38.4^{a}**			
	35 (W2)	37.15^{a}	37.2^{a}	38.8^{a}	39.0^{a}	**38.0^{ab}**			
	55 (W3)	36.9^{a}	37.3^{a}	36.8^{a}	35.3^{a}	**36.6^{b}**			
**MDL (%)**	75 (W4)	36.7^{a}	35.3^{a}	36.8^{a}	37.1^{a}	**36.5^{b}**			
	**Mean**	**37.2**	**37.1**	**37.6**	**37.5**	**37.4**	**0.52**	**ns**	**6.0**
	**SE**	**1.04**	**1.04**	**1.04**	**1.04**	**0.52**			
	**LSD.05**	**ns**	**ns**	**ns**	**ns**	**1.69**			
	**CV (%)**					**8.6**			**24.3**
**2021**	Control 20	41.9^{ab}	42.35^{ab}	42.2^{ab}	42.65^{ab}	**42.3^{a}**			
	35	41.35^{ab}	43.20^{ab}	44.15^{a}	42.73^{ab}	**42.9^{a}**			
	55	43.33^{ab}	43.23^{ab}	42.12^{ab}	41.25^{b}	**42.5^{a}**			
	75	41.35^{ab}	41.53^{ab}	42.87^{ab}	41.95^{ab}	**41.9^{a}**			
	**Mean**	**42.98**	**42.58**	**42.84**	**42.1**	**42.4^{a}**	**0.53**	**ns**	**4.7**
	**SE**	**1.07**	**1.07**	**1.07**	**1.07**	**0.53**			
	**LSD.05**	**ns**	**ns**	**ns**	**ns**	**ns**			
	**CV (%)**					**6.216**			
**2022**	Control 20	33.75^{a-e}	35.08^{ab}	34.20^{a-d}	34.85^{abc}	**34.47^{a}**			
	35	32.95^{a-e}	31.20^{def}	33.35^{a-e}	35.33^{a}	**33.21^{ab}**			
	55	30.48^{ef}	31.40^{c-f}	31.55^{b-f}	29.33^{f}	**30.69^{b}**			
	75	32.13^{a-f}	29.08^{f}	30.700^{def}	32.15^{a-f}	**31.01^{b}**			
	**Mean**	32.3	31.69	32.45	32.91	**32.3^{b}**	**0.69**	**ns**	**12.4**
	**SE**	1.38	1.38	1.38	1.38	**0.69**			
	**LSD.05**					**ns**			
	**CV (%)**					**17.4**			
**Significance of ANOVA result**									
**Y*MDL**		**ns**	**ns**	**ns**	**ns**	**ns**			
**Y*CRH**		**ns**	**ns**	**ns**	**ns**	**ns**			
**MDL*CRH**		**ns**	**ns**	**ns**	**ns**	**ns**			
**Y*MDL*CRH**		**ns**	**ns**	**ns**	**ns**	**ns**			

CV, coefficient of variation; LSD0.05, least significant difference at p = 0.05; SE, standard error; ns, non-significant; Y*MDL, interaction of year and moisture depletion level; Y*CRH, interaction of year and CRH; MDL*CRH, interaction of soil moisture depletion level and CRH; Y*MDL*CRH, interaction of year, soil moisture depletion level, and CRH; means connected with the same letter/s were not significantly different at p = 0.05.

3.4. Number of tillers per plant

The number of tillers per plant was significantly influenced by the main effect of soil moisture depletion level ($p<0.01$). However, the effect of CRH and the interaction with MDL were not significant on the NTPP of tef ($p > 0.05$). The maximum NTPP (1.71) was recorded from 55% MDL, but the value was indifferent as compared to 20% MDL, which provided a mean NTTP of 1.70. The fewest NTPP (1.20) was recorded from 35% MDL, but was not statistically different from 75% MDL (1.30). The variation in number of tillers per plant was in agreement with the result [60], who stated the difference in response of genotypes under optimal and sub-optimal moisture conditions in terms of the number of productive tillers.

Table 5.The effect of soil water depletion level and silicon application rate on tiller number of tef

**MDL (%)**	**CRH (kg ha^{-1})**							
	**0**	**291**	**582**	**873**	**Mean**	**SE**	**LSD.05**	**CV (%)**
20	1.65^{ab}	2.1^{a}	1.35^{b}	1.68^{ab}	**1.70^{a}**			
35	1.15^{b}	1.15^{b}	1.28^{b}	1.18^{b}	**1.20^{b}**			
55	2.2^{a}	1.55^{ab}	1.55^{ab}	1.58^{ab}	**1.71^{a}**			
75	1.23^{b}	1.15^{b}	1.65^{ab}	1.23^{b}	**1.30^{b}**			
**Mean**	**1.55**	**1.46**	**1.46**	**1.41**	**1.5**	**0.1265**	**ns**	**35.3**
**SE**					**0.1261**			
**LSD.05**					**0.25**			
**CV (%)**					**21.0**			
**MDL**	**	**	**ns**	**ns**	**			
**MDL*CRH**	**ns**	**ns**	**ns**	**ns**	**ns**			

Note that this result was only based on one-year data (2022); LSD.05, least significant difference at a probability level of 0.05; SE, standard error; MDL, soil moisture depletion level; CRH, carbonized rice husk; MDL*CRH, interaction of soil moisture depletion level and CRH; means connected with the same letter are not significantly different at the p = 0.05 level.

3.5. Grain and straw yields

The combined analysis over the two growing periods showed that soil moisture, silicon application rate, and their interaction effect on both GY and SY were not significant ($p<0.05$). However, the mean GY and SY were significantly higher ($p<0.001$) in 2021 than in 2022. In

2021, the mean yield from the main-plot treatments (MDL) ranged from 2279–2460 kg ha^{-1}, whereas in 2022, it was between 1169.8 and 1227.9 kg ha^{-1}. The overall mean GY measured in 2021 was higher than the GY in 2022 by about 99.7%. As compared to the on-farm mean national yield, the GY measured in 2021 showed a 39.4% increment, but in 2021, the GY decreased by 29.5%. Based on the result of COY, a similar trend was observed for tef SY. The mean SY in 2021 was 121.8% higher than the SY recorded in 2022.

Table 6. The effect of soil moisture depletion and the application of silicon on tef grain and straw yields of tef

Treatment		GY (kg ha^{-1})			SY (kg ha^{-1})		
		2021	2022	COY	2021	2022	COY
**MDL (%)**	Control (20)	2460.6^{a}	1177.0^{a}	1818.8^{a}	5168.7^{a}	2618.8^{a}	3893.8^{a}
	35	2318.0^{a}	1184.4^{a}	1751.2^{a}	5165.4^{a}	2381.5^{a}	3773.4^{a}
	55	2444.1^{a}	1227.9^{a}	1835.9^{a}	5388.0^{a}	2192.2^{a}	3790.1^{a}
	75	2279.2^{a}	1169.8^{a}	1724.5^{a}	5220.6^{a}	2248.1^{a}	3734.4^{a}
**CRH (0)**	Control (20)	2631.0^{a}	1185.5^{abc}	1908.2^{a}	5299.8^{ab}	2709.4^{ab}	4004.6^{a}
	35	2158.3^{b}	1237.9^{abc}	1698.1^{a}	5289.3^{ab}	2511.6^{abc}	3900.4^{a}
	55	2390.5^{ab}	1149.7^{bc}	1770.1^{a}	4940.3^{ab}	2083.3^{c}	3511.8^{a}
	75	2187.5^{ab}	1176.7^{bc}	1682.1^{a}	4554.0^{b}	2343.8^{abc}	3448.9^{a}
**CRH (291)**	Control (20)	2490.3^{ab}	1137.5^{bc}	1813.9^{a}	5119.0^{ab}	2491.1^{abc}	3805.0^{a}
	35	2260.0^{ab}	1108.2^{bc}	1684.1^{a}	4995.3^{ab}	2194.8^{bc}	3595.0^{a}
	55	2616.3^{ab}	1400.7^{a}	2008.5^{a}	5845.8^{a}	2215.5^{bc}	4030.6^{a}
	75	2225.5^{ab}	1089.8^{bc}	1657.6^{a}	5342.5^{ab}	2131.6^{c}	3737.1^{a}
**CRH (582)**	Control (20)	2419.8^{ab}	1177.2^{bc}	1798.5^{a}	5150.3^{ab}	2490.7^{abc}	3820.5^{a}
	35	2435.0^{ab}	1210.1^{abc}	1822.6^{a}	5175.0^{ab}	2425.7^{abc}	3800.4^{a}
	55	2320.0^{ab}	1062.3^{c}	1691.1^{a}	5517.3^{ab}	2139.9^{c}	3828.6^{a}
	75	2427.0^{ab}	1242.1^{abc}	1834.5^{a}	5657.8^{ab}	2263.1^{bc}	3960.4^{a}
**CRH (873)**	Control (20)	2301.5^{ab}	1208.0^{abc}	1754.7^{a}	5105.8^{ab}	2784.2^{a}	3945.0^{a}
	35	2418.8^{ab}	1181.3^{bc}	1800.0^{a}	5202.00^{ab}	2393.6^{abc}	3797.8^{a}
	55	2449.5^{ab}	1298.6^{ab}	1874.1^{a}	5248.8^{ab}	2329.9^{abc}	3789.4^{a}
	75	2276.8^{ab}	1170.6^{bc}	1723.7^{a}	5328.3^{ab}	2254.0^{bc}	3791.1^{a}
**Significance (ANOVA)**							
	**Year**			***			***
	**MDL**	**ns**	**ns**	**ns**	**ns**	**ns**	**ns**
	**CRH**	**ns**	**ns**	**ns**	**ns**	**ns**	**ns**
	**Y*CRH**			**ns**			**ns**
	**Y*MDL**			**ns**			**ns**
	**MDL*CRH**	**ns**	**ns**	**ns**	**ns**	**ns**	**ns**
	**CV (%)[1]**	**13.9**	**26.8**	**16.5**	**15.2**	**27.4**	**19.1**
	**CV (%)[2]**	**11.3**	**12.8**	**14.4**	**15.8**	**15.3**	**16.8**

[1,2] CV for the main (MDL) and sub-plot (CRH) factors; GY, grain yield; SY, straw yield; Y, year; MDL, soil moisture depletion level; CRH, carbonized rice husk; ANOVA, analysis of variance; CV, coefficient of variation; Y*MDL, interaction of year and soil moisture depletion level; Y*CRH, year, and CRH interaction; MDL*CRH, interaction of soil moisture depletion and CRH; *, **, ***, ns are significant at 0.05, 0.01, and 0.001 probability levels, respectively; ns is a non-significant difference ($p > 0.05$); Treatments within the group connected with the same letter were not significantly different.

The difference across the production season could be due to the variation in inter-annual crop management. The change in the rotating crop and the low input use cultivation during the

rainy season could have contributed to variation in GY and SY across seasons. The experimental land was covered by white lupine just prior to the 2021 tef experiment, whereas Niger seed was sown the sown as cover crop before the second season experiment. Researcher [61] stated that intra-season management including fertilization and crop rotation may also have had an influence on the significant difference over the two cropping years. In addition, irrigation was more frequent during 2021 and slightly less frequent in 2022 during the early stage of crop development.

3.6. Harvest index

The harvest index of tef was significantly (p<0.01) influenced by the change in the soil moisture depletion level. The effect of MDL was significantly (p<0.001) interacted with the cropping year. However, both the main effect of CRH and its interaction with MDL on HI of tef were not significant (p>0.05). The interaction of CHR with year was also not significant on the HI of tef. Both deficit and excessive soil moisture application levels had a significant effect on plant growth and development. Deficit irrigation causes stunted growth, which could reduce overall biomass yield, while increasing the harvest index of crops, while excessive application reduces respiration rate of plants [61]. The result was not in line with [62], who stated the sever effect of moisture stress during the late-season stage than early ones.

Table 7. The combined analysis result of two year experiment showing the effect of soil soil moisture depletion and the application of silicon on harvest index of tef

	Treatments	CRH (kg ha^{-1})							
		**0**	**291**	**582**	**873**	**Mean**	**SE**	**LSD.05**	**CV (%)**
**MDL (%)**	**Control (20)**	31.9^{bcd}	32.2^{bcd}	31.9^{bcd}	30.7^{d}	**31.7^{b}**	**0.42**		
	**35**	31.4^{d}	32.5^{a-d}	32.6^{a-d}	32.7^{a-d}	**32.2^{b}**	**0.58**		
	**55**	34.4^{ab}	35.0^{a}	31.8^{cd}	34.2^{abc}	**33.8^{a}**	**0.70**		
	**75**	32.9^{a-d}	31.9^{bcd}	32.9^{a-d}	32.3^{bcd}	**32.5^{b}**	**0.62**		
	**Mean**	**32.7**	**32.9**	**32.3**	**32.5**	**32.6**	**0.48**	**ns**	**8.1**
	**SE**	**0.55**	**0.65**	**0.61**	**0.62**	**0.48**			
	**LSD.05**	**ns**	**ns**	**ns**	**ns**	**1.12**			
	**CV (%)**					**6.6**			
**2021**	**Control (20)**	33.0a	33.0a	31.9a	31.3a	33.0a			
	**35**	29.6a	31.3a	32.0a	32.2a	29.6a			
	**55**	33.2a	30.9a	30.1a	32.2a	33.2a			
	**75**	32.5a	29.6a	30.4a	29.9a	32.5a			
	**Mean**	**32.1**	**31.2**	**31.1**	**31.4**	**32.1**	**0.72**	**ns**	**9.1**
	**SE**	**1.43**	**1.43**	**1.43**	**1.43**	**0.72**			
	**LSD.05**	**Ns**	**ns**	**ns**	**ns**	**ns**			
	**CV (%)**					**9.2**			
**2022**	Control (20)	30.8^{de}	31.4^{cde}	31.9^{cde}	30.2^{e}	31.1^{d}			
	**35**	33.2^{b-e}	33.7^{bcd}	33.3^{b-e}	33.2^{b-e}	33.3^{c}			
	**55**	35.6^{b}	39.1^{a}	33.4^{b-e}	36.2^{ab}	36.1^{a}			
	**75**	33.4^{b-e}	34.1^{bcd}	35.4^{b}	34.7^{bc}	34.4^{b}			
	**Mean**	**33.2**	**34.6**	**33.5**	**33.6**	**33.7**	**0.55**	**ns**	**7.2**
	**SE**	**1.1**	**1.1**	**1.1**	**1.1**	**0.55**			
	**LSD.05**	**ns**	*****	**ns**	**ns**	**0.68**			
	**CV (%)**					**2.5**			
**Significance of ANOVA**									
**Y*MDL**						*******			
**Y*CRH**		**ns**	**ns**	**ns**	**ns**	**ns**			
**MDL*CRH**		**ns**	**ns**	**ns**	**ns**	**ns**			
**Y*MDL*CRH**		**ns**	**ns**	**ns**	**ns**	**ns**			

CRH, carbonized rice husk; MDL, soil moisture depletion level; Y, year; SE, standard error; LSD.05, least significant difference at a probability of 0.05; CV, coefficient of variation; Y*MDL, Y*CRH, MDL*CRH, and Y*MDL*CRH are interactions of the defined factors; means connected by the same letter are not significantly different at a probability of 0.05.

3.7. Water use efficiency of tef

The COY result on the effect of MDL and CRH is presented in Table 8. The result showed that the WUE of tef was not significantly influenced both by the main and interaction effects of MDL and CRH ($p>0.05$). The COY result indicated that the interaction of MDL and CRH was only significantly affected tef WUE at $p<0.1$. This also interacted with the cropping year at the same level of significance. The individual year analysis result showed that in 2021, the interaction of MDL and CRH significantly affected the WUE of the tef crop ($p<0.05$). In 2022, however, the WUE of tef was not affected by the interaction of MDL and CRH, but it was significantly affected by the main effect of MDL ($p<0.05$). In 2021, the application of 582 kg CRH ha^{-1} combined with 75% of MDL showed a higher maximum WUE than any other treatment combination. In 2022, the maximum water use efficiency (0.55 kg ha^{-1} m^{-3}) was observed on 55% MDL but was not statistically different from 75% MDL, while the lowest WUE was recorded from the control (20% MDL) treatment, with a value of 0.4 kg ha^{-1} m^{-3}. Despite not being statistically different, the pooled mean values from the two-year data showed that 75% MDL had slightly been above all other treatments with a mean WUE of 1.997 kg ha^{-1} m^{-3}. The high water use efficiency value indicated a better grain yield with relatively little irrigation applied [61].

Table 8. The effect of soil water depletion and the application of silicon on tef water use efficiency (kg ha^{-1} m^{-3}) of tef

	**Treatments**	**CRH (kg ha^{-1})**							
		**0**	**291**	**582**	**873**	**Mean**	**SE**	**LSD.05**	**CV (%)**
**MDL (%)**	Control (20)	1.997a	1.992a	1.878a	1.824a	**1.923**	**0.23**		
	35	1.754a	1.833a	2.009a	1.881a	**1.869**	**0.17**		
	55	1.949a	2.182a	1.745a	2.112a	**1.997**	**0.17**		
	75	1.944a	2.030a	2.294a	1.724a	**1.998**	**0.20**		
	**Mean**	**1.91**	**2.00**	**1.98**	**1.89**	**1.947**	**1.12**	**ns**	**19.9**
	**SE**	**0.19**	**0.20**	**0.20**	**0.20**	**1.12**			
	**LSD.05**	**.**	**.**	**.**	**.**	**ns**			
	**CV (%)**								
**2021**	Control (20)	3.1^{ab}	2.96^{abc}	2.73^{a-d}	2.75^{a-d}	**2.9**			
	35 (W2)	2.22^{d}	2.35^{cd}	2.80^{a-d}	2.58^{a-d}	**2.5**			
	55 (W3)	2.496^{bcd}	2.96^{abc}	2.20^{d}	2.85^{a-d}	**2.6**			
	75 (W4)	2.48^{bcd}	2.76^{a-d}	3.25^{a}	2.23^{d}	**2.7**			
	**Mean**	**2.58**	**2.757**	**2.754**	**2.60**	**2.7**	**0.16**	**ns**	**18.1**
	**SE**	**0.31**	**0.31**	**0.31**	**0.31**	**0.16**			
	**LSD.05**	**0.694**	**ns**	**0.694**	**ns**	**ns**			
	**CV (%)**					**37.7**			
**2022**	Control (20)	0.342^{c}	0.409^{bc}	0.408^{bc}	0.360^{c}	**0.4^{b}**			
	35	0.514^{ab}	0.528^{ab}	0.470^{abc}	0.475^{abc}	**0.50^{a}**			
	55	0.561^{a}	0.560^{a}	0.521^{ab}	0.549^{ab}	**0.55^{a}**			
	75	0.563^{a}	0.522^{ab}	0.536^{ab}	0.486^{abc}	**0.53^{a}**			
	**Mean**	**0.495**	**0.504**	**0.48**	**0.47**	**0.49**	**0.07**	**ns**	**21.2**
	**SE**	**0.14**	**0.14**	**0.14**	**0.14**	**0.07**			
	**LSD.05**	*****	**ns**	**ns**	**ns**	**0.10**			
	**CV (%)**					**26.5**			
**Significance of ANOVA based on pooled mean over years**									
**Year**		**.**	**.**	**.**	**.**	**ns**			
**Y*MDL**						**ns**			
**Y*CRH**		**ns**	**ns**	**ns**	**ns**	**ns**			
**MDL*CRH**		**.**	**.**	**.**	**.**	**ns**			
**Y*MDL*CRH**		**.**	**.**	**.**	**.**	**ns**			
**CV (%)**						**38.4**			

* significant at $p<0.05$; (.), significant at $p<0.1$; MDL, water depletion level; CRH, carbonized rice husk; CV, coefficient of variation; LSD.05, least significant difference at the 0.05 level of probability; SE, standard error; Y*MDL, interaction of year and water depletion level; Y*carbonized rice husk, year and silicon rate; MDL*CRH, interaction of water depletion level and CRH; Y*MDL*CRH, interaction of year, water depletion level, and CRH.

3.8. Chlorophyll content

The results of the main and interaction effects of water depletion level and silicon rate on the chlorophyll content of tef leaf measured at four different periods of plant growth are presented in the table below (Table 9). The chlorophyll content of tef was significantly influenced by the level of soil water depletion at the first growth period ($p<0.05$). However, the effect was not significant for the remaining 3 sampling periods ($p > 0.05$). The main effect of CRH and its interaction with MDL was not significant for all the periods ($p>0.05$). The pooled mean result also showed that CC was not influenced by both the main and interaction effects of MDL and CRH. The result was based on single year data, which was collected in 2022. It could not tell us the consistency of the effect of treatments over the season. The significant effect of MDL during the early stage of the crop might be due to the crop's sensitivity to water stress during the early growth stage [14]. The non-significant treatment differences could be attributed to the more frequent application of irrigation during the early stages of crop emergence and development rather than an extended application interval. This was in agreement with [59], who stated the presence of significant tef response to irrigation frequency at the early, vegetative and productive stages.

Table 9. The main and interaction effect of water depletion level and CRH rate on the chlorophyll content of tef measured at different growth periods

**Treatments**	**levels**	**Chlorophyll content (nmol cm^{-2})**				
		**CC1**	**CC2**	**CC3**	**CC4**	**Mean**
**MDL (%)**	**Control (20)**	28.6a	31.92a	22.53b	6.83b	**22.47a**
	**35**	26.5b	32.26a	22.88b	8.13a	**22.43a**
	**55**	26.6b	32.28a	25.73a	7.96ab	**23.14a**
	**75**	28.6a	32.88a	23.29ab	7.11ab	**22.98a**
	**Mean**	**27.6**	**32.33**	**23.60**	**7.51**	**22.75**
	**LSD.05**	1.24	0.992	2.577	1.165	
**CRH (kg ha^{-1})**	**0**	27.72a	31.76ab	25.24a	7.08a	**22.95a**
	**291**	27.46a	32.99a	23.31ab	7.61a	**22.84a**
	**582**	27.56a	31.53b	22.58b	8.01a	**22.42a**
	**873**	27.54a	33.06a	23.29ab	7.33a	**22.80a**
	**Mean**	**27.57**	**32.33**	**23.60**	**7.51**	**22.75**
	**LSD.05**	1.714	1.395	2.312	1.36	**1.006**
**Significance (ANOVA)**						
	**MDL**	**	**ns**	**ns**	**ns**	**ns**
	**CRH**	**ns**	**ns**	**ns**	**ns**	**ns**
	**MDL*CRH**	**ns**	**ns**	**ns**	**ns**	**ns**
	**^{1}CV (%)**	**5.6**	**3.8**	**13.6**	**19.4**	**4.7**
	**CV (%)^{2}**	**8.7**	**6.0**	**13.7**	**25.3**	**6.2**

CC1, CC2, CC3, and CC4 are chlorophyll content measured 49, 64, 79, and 94 days after planting (period = 15 days); MDL, water depletion level; CRH, carbonized rice husk; ANOVA, analysis of variance; LSD.05, least significant difference at a probability level of 0.05; ^{1,2}CV, coefficient of variation for the main and sub-plot factors, respectively.

The trend of the chlorophyll content of tef leaf over time was shown using a line chart (Figure 4). The chart showed that CC had shown an increasing trend during the first period, followed by a decreasing trend for the remaining periods. The maximum CC of 28.62 and 27.7 nmol cm^{-2} was observed at 64 DAP, while the lowest CC of 6.8 and 7.1 nmol cm2 was observed at 94 DAP for both water depletion level and CRH application rate, respectively. This could be attributed to the change in plant physiological activity, whereby evapotranspiration and photosynthetic activities reached peaks at the end of the crop development stage and lows at the senescence stage [60].

Figure 4. Trend of tef leaf chlorophyll content (CC) over time (one season data average over treatments) as influenced by the main effect of water depletion level: 20, 35, 55, and 75% are water depletion levels, and b) CRH rate: 0,291, 582, and 873 kg ha^{-1}

4. Conclusion

Drought and lodging are among the major yield-limiting factors for tef cultivation in the highlands of Ethiopia. Tackling these problems through optimum irrigation and CRH supply could improve the yield and lodging resistance of Tef. Therefore, this study found that the water depletion level significantly influenced the plant height, number of tillers per plant, harvest index, and lodging resistance of tef but not the GY and SY. The effects of MDL and CRH and their interactions showed significant differences over the years. In the 2022 season, WUE was affected by the main effect of MDL, while the interaction effect of MDL and CRH was only significant ($p<0.1$). It was concluded that, though there was no significant difference in yield when tef was irrigated at 55% water depletion level, it provided a maximum HI of 33.8%, which was 2.1% more than the control, and increased the level of lodging resistance with a LI of 31.9%. Alternative silicon fertilizer sources, which are more economically effective, need to be considered in future research.

Acknowledgement

The authors are grateful to research and community service office, college of agriculture and environmental sciences, Bahir dar University for their relentless efforts to the successful implementation of the SENIT project.

References

1. Mahajan S, Tuteja N. Cold, salinity and drought stresses: An overview. Archives of Biochemistry and Biophysics. 2005.
2. Daryanto S, Wang L, Jacinthe PA. Global synthesis of drought effects on cereal, legume, tuber and root crops production: A review. Agric Water Manag. 2017;
3. Rockström J, Barron J, Fox P. Water productivity in rain-fed agriculture: challenges and opportunities for smallholder farmers in drought-prone tropical agroecosystems. In: Water productivity in agriculture: limits and opportunities for improvement. 2009.
4. Viste E, Korecha D, Sorteberg A. Recent drought and precipitation tendencies in Ethiopia. Theor Appl Climatol. 2013;
5. Haefele SM, Jabbar SMA, Siopongco JDLC, Tirol-Padre A, Amarante ST, Sta Cruz PC, et al. Nitrogen use efficiency in selected rice (Oryza sativa L.) genotypes under different water regimes and nitrogen levels. F Crop Res. 2008;
6. He M, Dijkstra FA. Drought effect on plant nitrogen and phosphorus: A meta-analysis. New Phytol. 2014;
7. Rouphael Y, Cardarelli M, Schwarz D, Franken P, Colla G. Effects of drought on nutrient uptake and assimilation in vegetable crops. In: Plant Responses to Drought Stress: From Morphological to Molecular Features. 2012.
8. Yihun, Y. M. *et al.* (2013) 'Crop Water Productivity of Irrigated Teff in a Water Stressed Region', *Water Resources Management*. doi: 10.1007/s11269-013-0336-x.
9. Zahoor SA, Ahmad S, Ahmad A, Wajid A, Khaliq T, Mubeen M, et al. Improving water use efficiency in agronomic crop production. Agron Crop Vol 2 Manag Pract. 2019;13–29.
10. Peng D, Chen X, Yin Y, Lu K, Yang W, Tang Y, et al. Lodging resistance of winter wheat (Triticum aestivum L.): Lignin accumulation and its related enzymes activities due to the application of paclobutrazol or gibberellin acid. F Crop Res. 2014;
11. Zhang Q, Zhang L, Evers J, Werf W van der, Zhang W, Duan L. Maize yield and quality in response to plant density and application of a novel plant growth regulator. F Crop Res. 2014;
12. Acreche MM, Slafer GA. Lodging yield penalties as affected by breeding in Mediterranean wheats. F Crop Res. 2011;

13. Baker CJ, Sterling M, Berry P. A generalised model of crop lodging. J Theor Biol. 2014;
14. Chanyalew S, Ferede S, Damte T, Fikre T, Genet Y, Kebede W, et al. Significance and prospects of an orphan crop tef. Planta. 2019.
15. Lyu X, Cheng Q, Qin C, Li Y, Xu X, Ji R, et al. GmCRY1s modulate gibberellin metabolism to regulate soybean shade avoidance in response to reduced blue light. Mol Plant. 2021;14(2):298–314.
16. Niu Y, Chen T, Zhao C, Zhou M. Improving crop lodging resistance by adjusting plant height and stem strength. Agronomy. 2021;11(12):2421.
17. Chu L Le, Yan Z, Sheng XX, Liu HQ, Wang QY, Zeng RF, et al. Citrus ACC synthase CiACS4 regulates plant height by inhibiting gibberellin biosynthesis. Plant Physiol. 2023;kiad159.
18. Wu Hao, Bai B, Li H. Multiple functions of gibberellin in modulating plant height, stem strength and drought tolerance. 2023;
19. Zhao J, Lai H, Bi C, Zhao M, Liu Y, Li X, et al. Effects of paclobutrazol application on plant architecture, lodging resistance, photosynthetic characteristics, and peanut yield at different single-seed precise sowing densities. Crop J. 2023 Feb 1;11(1):301–10.
20. Abdalla N, Taha N, Bayoumi Y, El-Ramady HR, Shalaby T. Paclobutrazol applications in agriculture, plant tissue cultures and its potential as stress ameliorant: A mini review. Environ Biodivers Soil Secur. 2021;5(2021):245–57.
21. Podgórska-Lesiak M, Sobkowicz P. Prevention of pea lodging by intercropping barley with peas at different nitrogen fertilization levels. F Crop Res. 2013;
22. Gong D, Zhang X, Yao J, Dai G, Yu G, Zhu Q, et al. Synergistic effects of bast fiber seedling film and nano-silicon fertilizer to increase the lodging resistance and yield of rice. Sci Rep. 2021;11(1):1–8.
23. Liu Y, Guo L, Gao Y, Wang Y. Effects of combined application of potassium and silicon fertilizer on carbohydrate and lodging resistance of oil flax stem. Chinese J Oil Crop Sci. 2022;44(2):410.
24. Rajput VD, Minkina T, Feizi M, Kumari A, Khan M, Mandzhieva S, et al. Effects of silicon and silicon-based nanoparticles on rhizosphere microbiome, plant stress and growth. Biology (Basel). 2021;10(8):791.
25. Olagunju SO, Atayese MO, Sakariyawo OS, Dare EO, Tang C. Effects of multi-growth

stage water deficit and orthosilicic acid fertiliser on lodging resistance of rice cultivars. Crop Pasture Sci. 2022;73(4):370–89.
26. Raza MAS, Zulfiqar B, Iqbal R, Muzamil MN, Aslam MU, Muhammad F, et al. Morpho-physiological and biochemical response of wheat to various treatments of silicon nano-particles under drought stress conditions. Sci Rep. 2023;13(1):2700.
27. Meena VD, Dotaniya ML, Coumar V, Rajendiran S, Ajay, Kundu S, et al. A case for silicon fertilization to improve crop yields in tropical soils. Proc Natl Acad Sci India Sect B - Biol Sci. 2014;84(3):505–18.
28. Gomaa MA, Kandil EE, El-Dein AAM, Abou-Donia MEM, Ali HM, Abdelsalam NR. Increase maize productivity and water use efficiency through application of potassium silicate under water stress. Sci Rep. 2021;11(1):1–8.
29. Crusciol CAC, Pulz AL, Lemos LB, Soratto RP, Lima GPP. Effects of silicon and drought stress on tuber yield and leaf biochemical characteristics in potato. Crop Sci. 2009;
30. Ma S, Duan A, Ma S, Yang S. Effect of early- stage regulated deficit irrigation on stem lodging resistance, leaf photosynthesis, root respiration and yield stability of winter wheat under post- anthesis water stress conditions. Irrig Drain. 2016;65(5):673–81.
31. Yan G chao, Nikolic M, YE M jun, XIAO Z xi, LIANG Y chao. Silicon acquisition and accumulation in plant and its significance for agriculture. Journal of Integrative Agriculture. 2018.
32. Savant NK, Korndörfer GH, Datnoff LE, Snyder GH. Silicon nutrition and sugarcane production: A review. J Plant Nutr. 1999;
33. Bosnic P, Bosnic D, Jasnic J, Nikolic M. Silicon mediates sodium transport and partitioning in maize under moderate salt stress. Environ Exp Bot [Internet]. 2018;155(June):681–7. Available from: https://doi.org/10.1016/j.envexpbot.2018.08.018
34. Maillard A, Ali N, Schwarzenberg A, Jamois F, Yvin JC, Hosseini SA. Silicon transcriptionally regulates sulfur and ABA metabolism and delays leaf senescence in barley under combined sulfur deficiency and osmotic stress. Environ Exp Bot [Internet]. 2018;155(June):394–410. Available from: https://doi.org/10.1016/j.envexpbot.2018.07.026
35. Ahmed M, Fayyaz-ul-Hassen, Khurshid Y. Does silicon and irrigation have impact on

drought tolerance mechanism of sorghum? Agric Water Manag. 2011;

36. Cuong TX, Ullah H, Datta A, Hanh TC. Effects of Silicon-Based Fertilizer on Growth, Yield and Nutrient Uptake of Rice in Tropical Zone of Vietnam. Rice Sci. 2017;24(5):283–90.
37. Epstein E. The anomaly of silicon in plant biology. Proceedings of the National Academy of Sciences of the United States of America. 1994.
38. Liang Y, Nikolic M, Bélanger R, Gong H, Song A. Silicon in agriculture. LIANG, Y al Silicon-mediated Toler to salt Stress Springer Sci. 2015;123–42.
39. Satbaev B, Yefremova S, Zharmenov A, Kablanbekov A, Yermishin S, Shalabaev N, et al. Rice husk research: From environmental pollutant to a promising source of organo-mineral raw materials. Materials (Basel). 2021;14(15):4119.
40. Shen Y, Zhao P, Shao Q. Porous silica and carbon derived materials from rice husk pyrolysis char. Microporous Mesoporous Mater. 2014;188:46–76.
41. Moayedi H, Aghel B, Nguyen H, Rashid ASA. Applications of rice husk ash as green and sustainable biomass. J Clean Prod. 2019;237:117851.
42. Koyama S, Hayashi H. Rice yield and soil carbon dynamics over three years of applying rice husk charcoal to an Andosol paddy field. Plant Prod Sci. 2017;20(2):176–82.
43. Gelaw AM, Qureshi AS. Tef (Eragrostis tef): a superfood grain from Ethiopia with great potential as an alternative crop for marginal environments. Emerg Res Altern Crop. 2020;265–78.
44. Barretto R, Buenavista RM, Rivera J Lou, Wang S, Prasad PVV, Siliveru K. Teff (Eragrostis tef) processing, utilization and future opportunities: a review. Int J Food Sci Technol. 2021;56(7):3125–37.
45. Sridhara S, Punith Gowda HN, Manoj KN, Gopakkali P. Nutritional importance of Teff (Eragrostis tef (Zucc.) Trotter) and human health: a critical review. Int Sch J. 2021;11(2).
46. Hoogenboom G, Porter CH, Boote KJ, Shelia V, Wilkens PW, Singh U, et al. Advances in crop modeling for a sustainable agriculture. DSSAT Crop Model Ecosyst. 2019;173–216.
47. Gomez KA, Gomez AA. Statistical procedures for agricultural research. John wiley & sons; 1984.
48. MANR (2016) ‘plant variety release. Protection and seed 0ual1ty control directorate’.

Ministry of Agriculture and Natural Resources, p. 330.
49. Tesfahun W. Tef Yield Response to NPS Fertilizer and Methods of Sowing in East Shewa, Ethiopia. J Agric Sci. 2018;13(2):162.
50. Team, R. core. (2021). *R: A Language and Environment for Statistical Computing* (4.1.1). R Foundation for Statistical Computing. https://www.r-project.org/
51. Hanusz, Z., & Tarasińska, J. (2015). Normalization of the Kolmogorov–Smirnov and Shapiro–Wilk tests of normality. *Biometrical Letters*, *52*(2), 85–93.
52. Liang YC, Ma TS, Li FJ, Feng YJ. Silicon availability and response of rice and wheat to silicon in calcareous soils. Commun Soil Sci Plant Anal. 1994;25(13–14):2285–97.
53. Korndörfer GH, Lepsch I. Effect of silicon on plant growth and crop yield. Stud Plant Sci. 2001;
54. Ding TP, Zhou JX, Wan DF, Chen ZY, Wang CY, Zhang F. Silicon isotope fractionation in bamboo and its significance to the biogeochemical cycle of silicon. Geochim Cosmochim Acta. 2008;
55. Masulili A, Utomo WH, MS S. Rice Husk Biochar for Rice Based Cropping System in Acid Soil 1. The Characteristics of Rice Husk Biochar and Its Influence on the Properties of Acid Sulfate Soils and Rice Growth in West Kalimantan, Indonesia. J Agric Sci. 2010;
56. Yanai J, Taniguchi H, Nakao A. Evaluation of available silicon content and its determining factors of agricultural soils in Japan. Soil Sci Plant Nutr. 2016;
57. Ligaba-Osena A, Guo W, Choi SC, Limmer MA, Seyfferth AL, Hankoua BB. Silicon Enhances Biomass and Grain Yield in an Ancient Crop Tef [Eragrostis tef (Zucc.) Trotter]. Front Plant Sci. 2020;11(November):1–15.
58. Ullah, H. *et al.* (2019) 'Improving water use efficiency, nitrogen use efficiency, and radiation use efficiency in field crops under drought stress: A review', *Advances in agronomy*, 156, pp. 109–157.
59. Fikre T, Genet Y, Kebede W, Tolossa K, Chanyalew S, Demissie M, et al. Yield and Agronomic Performance of Selected Semi-dwarf Tef (Eragrostis tef (Zucc.) Trotter) Genotypes under Irrigation Farming System in Ethiopia. Am J Plant Biol. 2020;5(4):110–9.
60. Abraha MT, Hussein S, Laing M, Assefa K. Genetic variation and trait association of tef [*'Eragrostis tef'*(Zucc.) Trotter] evaluated under optimal and moisture stressed

environments. Aust J Crop Sci. 2017;11(3):241–7.

61. Zhao W, Liu L, Shen Q, Yang J, Han X, Tian F, et al. Effects of water stress on photosynthesis, yield, and water use efficiency in winter wheat. Water. 2020;12(8):2127.
62. Teferra T, Tefera H, Simane B, Tuinstra M. The influence of drought stress on yield of tef (Eragrostis tef. Trop Sci. 2000;40(1):40–5.

Supporting information

S1 Fig. Preparation procedure for carbonized from a well dried rice husk: Phase I to III shows heating process and change in the color of the husk yellow to black; phase IV is the final stage indicated by complete conversion of rice husk to black color, thus cooling takes place by spraying water to avoid conversion to the ash form

S2 Fig. Field layout of a two factor experiment in a split-plot design

S3 Fig. Broadcast incorporation of carbonized rice husk: a) application by broadcasting; b) manual mixing up with the soil

Figure Click here to access/download;Figure;Fig 1.tiff

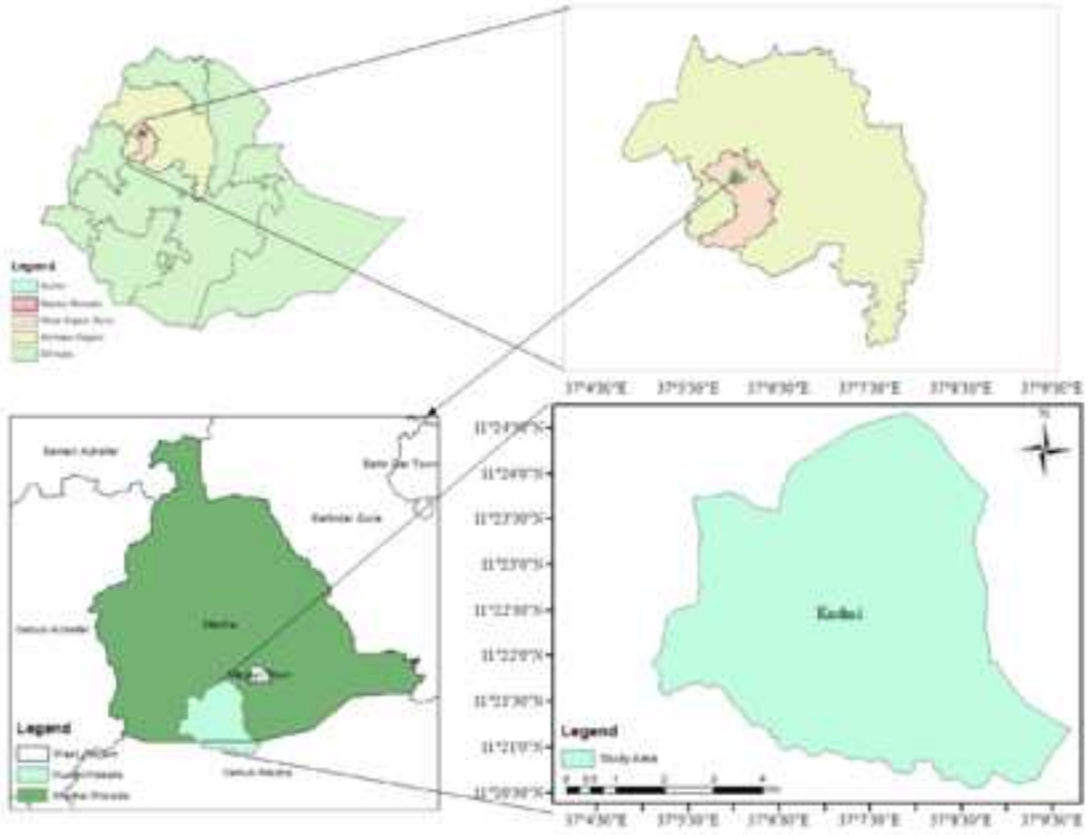

Fig 1. Map of the study site

Figure

Click here to access/download;Figure;Fig 2.tiff

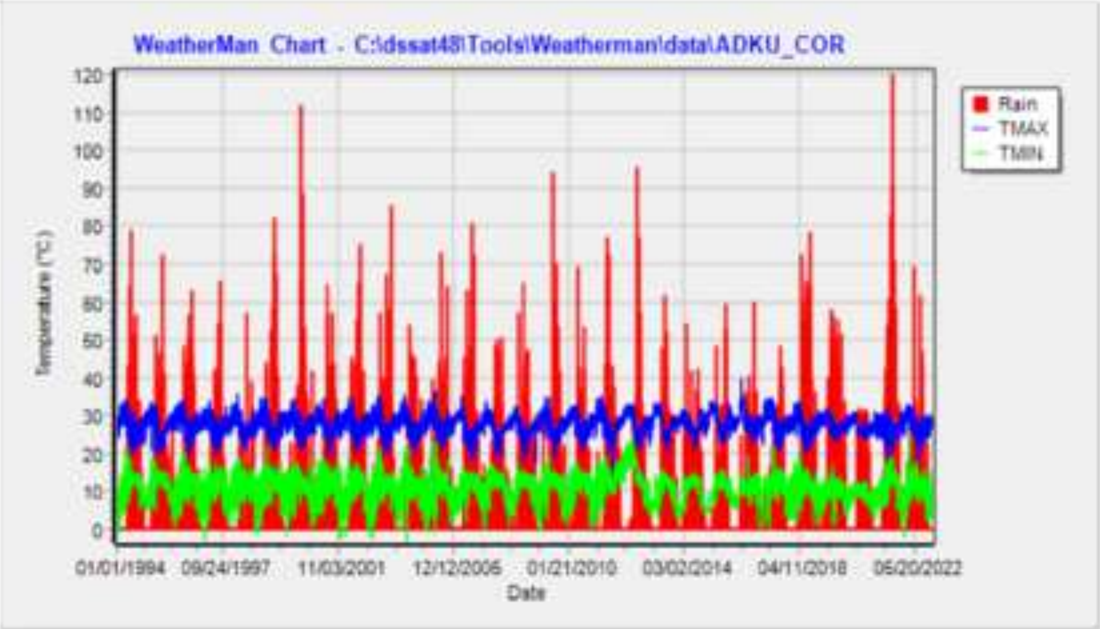

Fig 1. Mean daily minimum and maximum temperatures and rain weather data for 01/01/1994 to 31/12/2022 (Source: National Meteorological Agency of Ethiopia, Merawi Agro-meteorological station)

Figure Click here to access/download;Figure;Fig-3.tiff

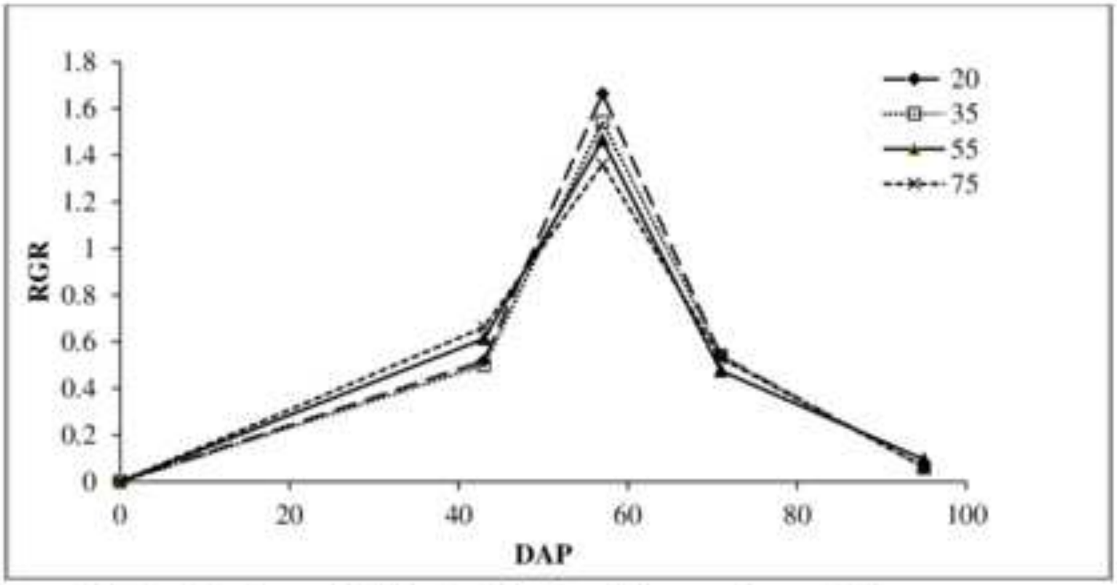

Figure 1. The effect of water depletion level on the relative growth rate of tef

 Click here to access/download;Figure;Fig-4.tiff

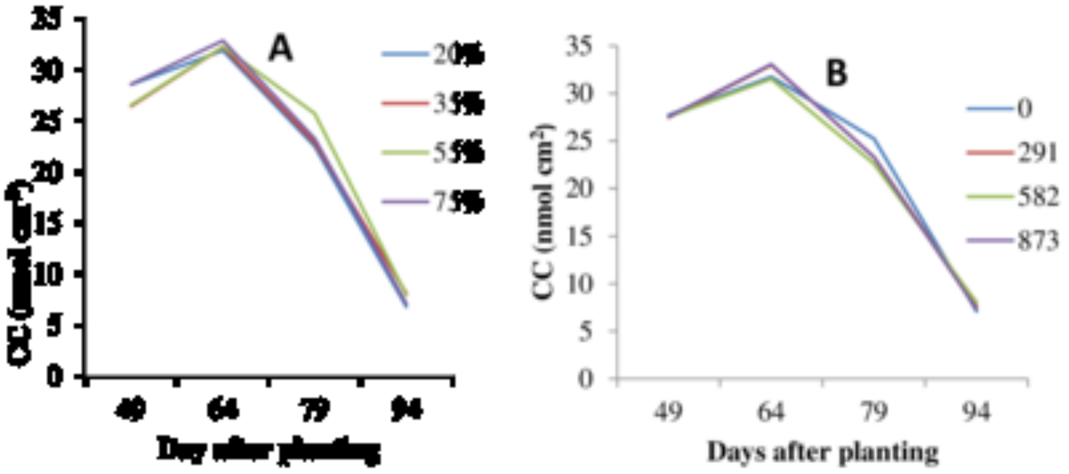

Figure 4. Trend of tef leaf chlorophyll content (CC) over time (one season data average over treatments) as influenced by the main effect of water depletion level: 20, 35, 55, and 75% are water depletion levels, and b) CRH rate: 0,291, 582, and 873 kg ha^{-1}

Click here to access/download
Supporting Information
S1-Fig.tiff

Click here to access/download
Supporting Information
S2-Fig.tiff

Click here to access/download
Supporting Information
S3-Fig.tiff
