# Supplementary material for: Yield, lodging, and water use efficiency of Tef [Eragrostis tef (zucc) Trotter] in response to carbonized rice husk application under variable moisture condition
Source: PLoS One. 2024 Mar 7;19(3):e0298416. doi: 10.1371/journal.pone.0298416 (PMC10919715; doi:10.1371/journal.pone.0298416)
Supplement: Attachment — Submitted filename: Response to Reviewers.docx [file pone.0298416.s007.docx]

**Response to Reviewers**

First of all we the authors are very grateful to all the reviewers for their valuable and detailed comments, suggestion, and questions made for the betterment of the paper. Here we tried to address the questions raised by the reviewers, while accepting all the comments.

a. **Response to Academic Editors**

#1. Regarding style of writing: we did our best to revise and ensure the requirement of the style of writing of the journal

#2. About grant information: this research was conducted no formal funding other than labor and equipment supplies. So, we would like to make correction on the financial disclosure.

#3. Repository information is included here with the revised manuscript

#4. About the map (Fig 1) which may be copyrighted: the map was created using arcGIS software for illustration purpose. So, we removed it by indicating the site with projection points in the text.

#5. Figures S1 and S3 are the original photos, which were taken during the research work and are original, which are owned by the corresponding author, thus we affirm that there is no copy right issue.

b. **Responses to Reviewer (R#1)**

**Q#1.** What do the terms LAI, LI, PH, and NTPP stands for?

**Authors’ Response:** We missed to define the abbreviations, which leads to confusion of the readers and we accepted and made a mistake and took correction.

**Q#2.** Does this mean that the different CRH levels were combined as a treatment?

**Authors’ Response:** No. It was typing error. CRH levels were combined with moisture depletion levels as a treatment. Therefore, we made a correction on the statement.

**Q#3.** From which treatment does the lowest lodging index (31.9%) was recorded?

**Authors’ response:** we found that the statement that we wrote lacked clarity and needs restructuring. Therefore, we made correction on the conclusion.

**Q#4.** How did the authors arrive at this suggestion (alternative silicon fertilizer sources) in the context of the current study?

**Authors’ response:** we arrived at this suggestion, based on the market analysis of for the rice husk, loading and unloading, and its preparation, the application of carbonized rice husk could be expensive to the farmers in the study site, and we believed to see other economically feasible silicon sources. However, it looks rubbish to suggest this in the context of the current study and we have changed it.

**Q#5.** What does the 22% yield loss comprised off?

**Authors’ Response:** The 22% yield loss comprises the grain yield.

**Q6.** How does silicon helps in achieving increased P availability and avoid heavy metal toxity?

Silicon increases P availability by increasing root exudation of organic acids that mobilize Pi in the rhizosphare and up-regulated Pi transporters

**Q7.** Were the levels of carbonized rice husk based on the silicon content?

**Authors’ response:** No. the levels of carbonized rice husk were set based on the recommendation provided for rice crop.

**Q8.** Why was the water depletion level and the CRH treated as the fixed variable and replication as random variable and not the other way round?

**Authors’ response:** According to Gomez and Gomez (1984), in an experimental research there are two variations, which are the within group and between group variations. The treatments are the variables that should be under the researchers control and levels are predefined at a fixed level, thus helps to detect the real variation comes from the between group variation ((fixed effect), replication is required to control the random effect that comes from the within group variation and helps to estimate the magnitude of the random error (random effect) not the treatment effect.

**Q9.** Regarding tables or use bar charts

For the majority of the tables, which contain both the main and interaction effects of MDL and CRH we tried to make them as simple as possible in the revised paper, additionally we use bar chart for the analysis result of chlorophyll content and its trend over growing periods.

Q10. What does COY stands for?

Authors’ Response: COY stands for combined analysis over the years

c. **Responses to Reviewer (R#2)**

With due respect the reviewer provided comments and suggestion to be made, thus we accept those comments and suggestion provided to us and included to this revised manuscript.
